# Babesiosis Acquired through Blood Transfusion, California, USA

**DOI:** 10.3201/eid1505.081562

**Published:** 2009-05

**Authors:** Van Ngo, Rachel Civen

**Affiliations:** Los Angeles County Department of Public Health, Los Angeles, CA, USA

**Keywords:** Vector-borne infections, babesiosis, Babesia microti, blood transfusion, dispatch

## Abstract

Babesiosis was reported in a California resident who received a transfusion of blood products collected in the disease-endemic northeastern region of the United States. Babesiosis should be considered year-round in the diagnosis of febrile and afebrile patients with abnormal blood cell counts who have received blood products from disease-endemic areas.

Babesiosis is an infection of red blood cells (RBCs) caused by various species of the protozoan genus *Babesia*. Most human infections reported in the United States are attributed to *B. microti* and occur most frequently in the Northeast and less commonly in the Midwest (*1*). Infrequently, babesiosis cases have been documented in California and Washington; however, these cases were caused by local *Babesia*-like isolates, including *B. duncani* and a *B. divergens*–like parasite (*1**–**3*). *B. microti* infection is often asymptomatic but can potentially be severe and even fatal, especially in the elderly, asplenics, and other immunosuppressed persons. Symptoms can be nonspecific, mimicking many systemic infectious diseases, and include fever, chills, myalgias, fatigue, and jaundice caused by hemolytic anemia (*1*).

Babesiosis is transmitted primarily through the bite of an infected tick, typically *Ixodes* spp., although occasionally transmission occurs via transfusion of blood products collected from asymptomatic infected donors (*1*). More than 50 transfusion-related cases have been reported in the United States (*4*). This report describes a transfusion-acquired case of babesiosis caused by *B. microti* in a resident of Los Angeles County, California.

## The Case

On February 12, 2007, a 58-year-old man with metastatic esophageal cancer was admitted to an acute care facility for evaluation of hematemesis and normocytic anemia. The initial examination showed he had hypotension without fever, joint swelling, headaches, or rash. Laboratory evaluation showed a hemoglobin concentration of 8.4 mg/dL, a platelet count of 71,000/mm^3^, and a leukocyte count of 3.5 × 10^3^/mm^3^ with 19% bands. Results of liver function tests showed mild elevations in levels of aspartate transaminase (202 mg/dL), alanine transaminase (33 mg/dL), and total bilirubin (0.7 mg/dL).

An abnormal blood cell count prompted a manual differential count. *Babesia* spp. was identified on a peripheral smear and subsequently confirmed at the Los Angeles County Public Health Laboratory. The result of PCR analysis performed by a commercial laboratory was positive and highly specific for *B. microti* DNA, a result confirmed by the Centers for Disease Control and Prevention (CDC) (Table). The commercial laboratory also performed indirect fluorescent antibody (IFA) testing for *B. microti* and found both acute and convalescent specimens to be negative. Confirmatory testing at CDC corroborated the negative result for the acute specimen but showed the convalescent specimen, collected 8 days after onset, to be positive for *B. microti*, with a total antibody titer of 64. The patient was treated with azithromycin and atovaquone for 7 days, given 2 blood transfusions for anemia, and discharged in stable condition on February 16, 2007.

**Table:** Results of serologic testing and PCR analyses of specimens collected from a California resident with babesiosis and donors of packed red blood cells, 2007*

Specimen source	Residence	Date of transfusion†	Date of specimen collection	*B. microti* test results	
				PCR	IFA
Patient	California	–	Feb 12	Positive	<8‡
			Feb 20	–	64
Donor 1	Maine	Jan 1	Feb 26	Negative	256
Donor 2	Maine	Jan 1	Feb 26	–	<8
Donor 3	California	Jan 22	Feb 21	–	<8
Donor 4	California	Jan 23	Feb 22	–	<8
Donor 5	California	Jan 24	Feb 21	–	<8
Donor 6	California	Jan 24	Feb 21	–	<8

*IFA, immunofluorescent antibody. †Involving blood products from specified donor. ‡<8 is considered a negative titer for total *B. microti* antibody. IFA tests conducted at the Centers for Disease Control and Prevention Reference Diagnostic Laboratory.

Before admission, the patient had visited an oncology clinic numerous times for treatment related to his esophageal cancer: radiation therapy in October 2006, 3 chemotherapy courses from October 2006 through February 2007, and blood transfusions in January 2007. The patient received 6 units of packed red blood cells (PRBCs) and 2 units of fresh frozen plasma (FFP) over several clinic visits on January 1 and January 22–24, 2007.

The patient was in Salt Lake City, Utah, from January 13 through January 20, 2007; however, because of poor health, he did not engage in any outdoor activities. At least a year before his admission in 2006, the patient visited an undeveloped property near Klamath Falls, Oregon, where he spent time outdoors. He could not recall ever incurring a tick bite, seeing ticks, or having any animal contact.

The Table summarizes the serologic and PCR results for specimens collected from the patient and 6 PRBC donors. The PRBC units came from 2 blood banks: 1 in Maine (2 units) and 1 in California (4 units). A blood donor from Maine tested positive for *B. microti* by IFA, with a total antibody titer 256, but tested negative *B. microti* by PCR. Testing of specimens from remaining PRBC donors yielded negative results. Specimens from FFP donors were not tested because of the low risk for *Babesia* spp. transmission associated with plasma products.

The implicated donor was a 49-year-old male resident of Maine, where babesiosis is less common than in other states in the northeast. For example, whereas Maine typically reports <12 cases annually, Rhode Island has reported up to 61 cases (*5**,**6*). However, the donor resided in the southern coastal region of the state, where Maine’s cases are concentrated (*5*). He frequented tick-infested areas and is likely to have become infected in late August 2006, when he sought treatment for fever, chills, weight loss, and fatigue and was tested for various infections, including Lyme disease and ehrlichiosis. At that time, he was not tested for babesiosis. His health improved without a specific diagnosis or treatment, and he remained asymptomatic, but he evidently was parasitemic when he donated blood on December 20, 2006. Blood products from this donation were included in the transfusion the patient received on January 1, 2007. Between October 2005 and the blood donation on December 20, 2006, the donor made 3 donations.

All other recipients of blood products from this donor were residents of Maine. They were notified of the need for serologic testing; none were reported to be infected (V. Rea, pers. comm.).

## Conclusions

Babesiosis was documented in a man with metastatic cancer who resided in an area nonendemic for *B. microti*. On the basis of laboratory and epidemiologic information, we concluded that the patient acquired the infection via transfusion of infected PRBCs donated in a disease-endemic area thousands of miles away. The 12-day period from donation to transfusion was within the maximum 35 days that *B. microti* has been known to remain viable in refrigerated blood (*7*). The period from time of transfusion exposure until positive smear was ≈6 weeks; incubation periods for transfusion-related cases have ranged from weeks to many months (B. Herwaldt, pers. comm.). We had tentatively hypothesized that the patient might have acquired the infection in Oregon, where he spent substantial time outdoors, but remained asymptomatic until he became ill with cancer; however, we rejected this hypothesis because human cases of babesiosis have never been documented in Oregon (or in Utah where the man also visited) and because infections acquired in western states are more likely to be caused by local *Babesia* agents.

This case demonstrates that, even among transfused patients who show atypical symptoms of babesiosis, the possibility of infection should be considered if they have received blood products from disease-endemic areas and display abnormal blood cell counts, such as low iron and low leukocyte counts. Generalized debilitation associated with cancer and chemotherapy may have masked *Babesia*-related symptoms in this patient and undermined his immune response. This case also underscores the widening seasonal and geographic boundaries of babesiosis. Tick-borne babesiosis usually peaks from July through September (*4*), but because asymptomatic *Babesia* infection can persist for months to years, especially in untreated persons (*7**,**8*), transfusion-associated infection can occur throughout the year. Geographic limitations in babesiosis are virtually erased by the mobility of donors and blood products. The blood bank involved in this case has blood collection centers in California and Maine but provides blood products to hospitals throughout southern California and the East Coast. Medical evaluation for babesiosis in both febrile and afebrile transfusion patients should include a Giemsa-stained thin blood smear, an acute serologic evaluation by IFA testing and a convalescent serologic evaluation by IFA testing taken 4–6 weeks apart (*9*), and PCR evaluation of whole blood.

The varied clinical spectrum of babesiosis makes its detection in blood donors challenging. This case exemplifies the limitations in screening healthy asymptomatic donors for babesiosis. Available screening tests to detect *Babesia* spp. postdonation are not cost-effective and have inadequate sensitivity (*7**,**10*). Nucleic acid testing and inactivation procedures may provide useful options for detecting *Babesia* spp. in the future (*7**,**11*). Until effective screening procedures are available, however, diagnosis of babesiosis in blood donors will continue to be based primarily on clinical observation.
